# Treatment outcomes in NTM-PD in a high TB burden context

**DOI:** 10.5588/ijtldopen.24.0413

**Published:** 2024-12-01

**Authors:** S. Kang, J.E. Schmidt, I. Chen, S. Tiberi

**Affiliations:** ^1^ GSK, Beijing, China;; ^2^ GSK, Siena, Italy;; ^3^ GSK, Shanghai, China;; ^4^ GSK, Brentford, UK;; ^5^ Blizard Institute, Barts and The London School of Medicine and Dentistry, Queen Mary University of London, London, UK.

**Keywords:** antimicrobial treatment, China, clinical outcomes, non-tuberculous mycobacterial pulmonary disease, non-tuberculous mycobacteria

## Abstract

**BACKGROUND:**

Non-tuberculous mycobacterial (NTM) pulmonary disease (PD) is a significant concern in China, compounding the existing burden of TB. This review aims to summarise the treatment outcomes for NTM-PD in China.

**METHODS:**

We reviewed the evidence on NTM-PD, including treatment regimens and clinical outcomes, from 17 studies identified through screening of three Chinese biomedical databases.

**RESULTS:**

Antimicrobial treatment showed a microbiological cure rate ranging from 17.2% to 60.0% in studies with ≥50 NTM-PD patients, with lower rates observed among older and malnourished patients. The *Mycobacterium chelonae* abscessus group (MC-AG) and *Mycobacterium avium*-intracellulare complex (MAC) were the most prevalent NTM species in China. Higher microbiological cure rates were seen in MAC PD compared with MC-AG PD. The addition of cefoxitin and linezolid improved culture conversion rates in MC-AG-infected patients. One study (*n* = 24) demonstrated that resecting lesions and chemotherapy led to more favourable clinical outcomes.

**CONCLUSION:**

Treatment regimens recommended in Chinese guidelines yielded poor-to-moderate outcomes for NTM-PD in China, highlighting the need for further research into alternative antimicrobial treatments to improve efficacy.

## RÉSUMÉ

**CONTEXTE:**

Les maladies pulmonaires (PD) à mycobactéries non tuberculeuses (NTM) représentent un enjeu majeur en Chine, aggravant le poids de la TB. Cette analyse a pour objectif de synthétiser les résultats du traitement de la NTM-PD en Chine.

**MÉTHODES:**

Nous avons examiné les données concernant les NTM-PD, en tenant compte des protocoles de traitement et des résultats cliniques, dans 17 études sélectionnées à partir de trois bases de données biomédicales chinoises.

**RÉSULTATS:**

Le traitement antimicrobien a révélé un taux de guérison microbiologique variant entre 17,2% et 60,0% dans les études impliquant au moins 50 patients atteints de NTM-PD, des taux plus bas étant notés chez les patients âgés et souffrant de malnutrition. En Chine, les espèces de NTM les plus courantes étaient le groupe *Mycobacterium chelonae* abscessus (MC-AG) et le complexe *Mycobacterium avium-*intracellulare (MAC). Des taux de guérison microbiologique supérieurs ont été observés dans les cas de MAC-PD par rapport à ceux de MC-AG-PD. L'ajout de céfoxitine et de linézolide a contribué à améliorer les taux de conversion des cultures chez les patients infectés par le MC-AG. Une étude (*n* = 24) a montré que la résection des lésions, associée à une chimiothérapie, entraînait des résultats cliniques plus positifs.

**CONCLUSION:**

Les protocoles de traitement suggérés dans les recommandations chinoises ont produit des résultats allant de médiocres à modérés pour les NTM-PD en Chine, ce qui met en évidence l'importance de continuer les investigations sur des traitements antimicrobiens alternatifs afin d'en accroître l'efficacité.

Non-tuberculous mycobacteria (NTM) are widely distributed in the environment. Environmental exposure to NTM can cause infection and disease, primarily affecting immunocompromised patients and those with underlying structural lung diseases. Since the 1990s, NTM pulmonary disease (NTM-PD) has been a growing concern worldwide, as its incidence has risen in the United States and other countries with a low or moderate prevalence of TB.^1,2^

The increasing prevalence of NTM is a major concern, especially in China, which has a high prevalence of TB. The national TB epidemiology surveys conducted in 1979, 2000, and 2010 recorded rising rates of NTM isolates in sputum culture (4.3%, 11.1%, and 22.9%, respectively).^3^ A 2022 study on the prevalence of NTM isolates in respiratory samples positive for acid-fast bacilli (AFB) reported a high NTM isolation rate of 36.7% in Guangzhou between 2018 and 2021.^4^ Additionally, NTM-PD prevalence of 5.0% to 8.4% was observed in susceptible populations with underlying bronchiectasis.^5,6^ The most dominant NTM species are *Mycobacterium abscessus* complex (MABC) and *Mycobacterium avium*-intracellulare complex (MAC) in mainland China,^7,8^ with MABC prevailing in China (∼20% of total NTM cases) compared to other regions worldwide.^9^

In light of the changing epidemiology, the Chinese Academy of Medical Sciences published the expert consensus for diagnosis and treatment of NTM infection in 2012,^10^ which was updated and formulated in the 2020 guidelines.^11^ The updated 2020 edition detailed the treatment principles outlined in the 2012 edition. Both editions are overall consistent with the American Thoracic Society/Infectious Disease Society of America (ATS/IDSA) statement in 2007^12^ and the ATS, European Respiratory Society (ERS), European Society of Clinical Microbiology and Infectious Diseases (ESCMID), and IDSA clinical practice guideline in 2020,^13^ regarding diagnostic criteria and treatment recommendations.

The standard of care for NTM-PD in China is a combination therapy of 3–6 antimicrobials for a duration of 18–24 months or a minimum of 12 months after the conversion of cultures. The antimicrobial regimen depends on the causative species and the drug susceptibility test results, leading to subsequent modification in the treatment protocol. The dose, route of administration, and treatment duration are based on the use of these drugs in TB and other infections. As a result, the current treatment of NTM-PD is empirical and varies significantly.

**Figure 1.**
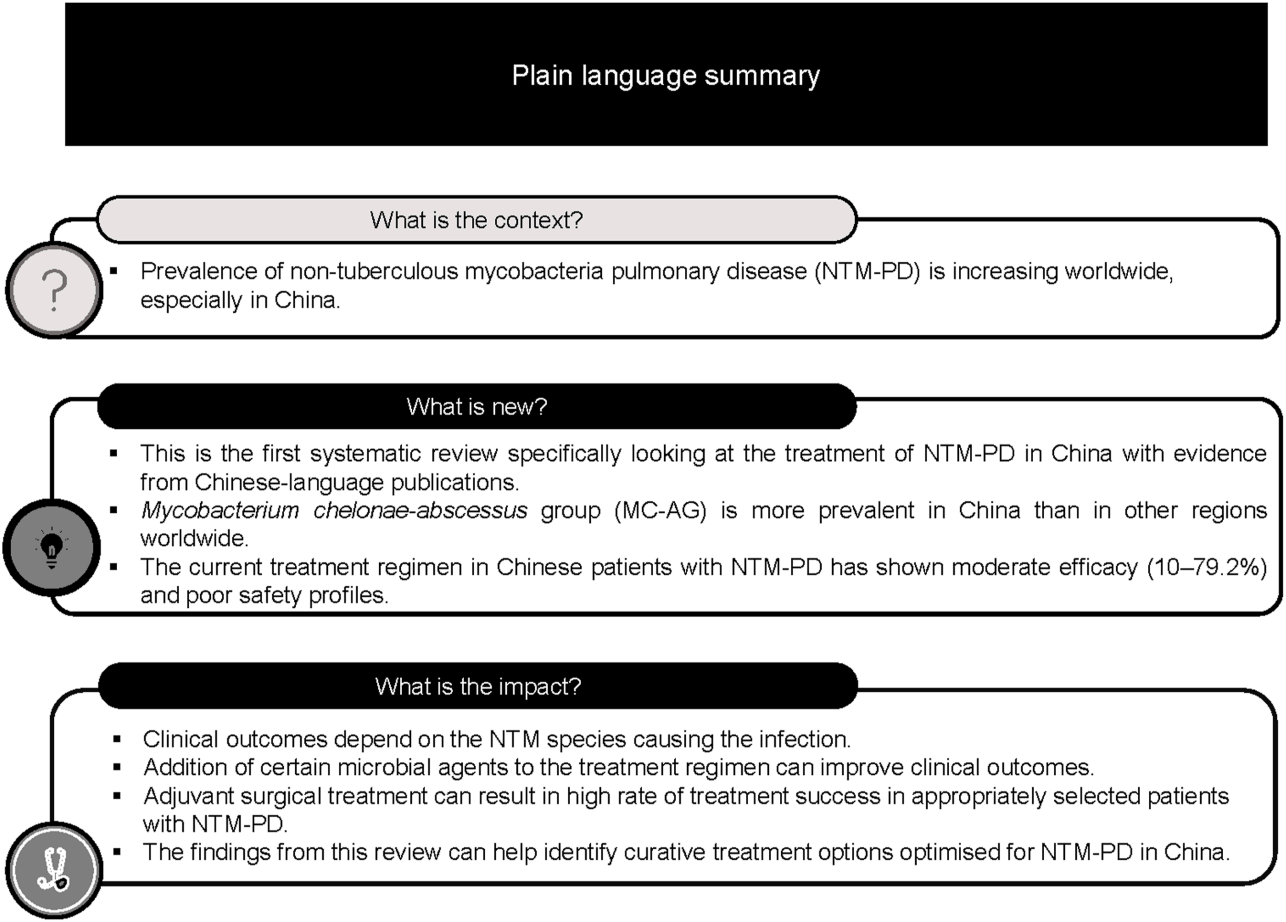
Plain language summary.

Most published studies were based on small patient groups and lacked an overview of the treatment for NTM and corresponding outcomes. Therefore, we aimed to summarise the NTM-PD treatment outcomes across China using a systematic literature review and meta-analysis approach following the Preferred Reporting Items for Systematic Reviews and Meta-Analyses (PRISMA) reporting standards for medical and epidemiological evidence syntheses.^14^ Figure 1 summarises this study’s context, outcomes, and impact on healthcare professionals.

## METHODS

### Data sources and search strategy

We conducted a retrospective, observational study using three Chinese biomedical bibliographic databases – China National Knowledge Infrastructure (CNKI), Wanfang Database, and Chinese Science and Technology Journals Database (VIP-CSTJ). Records published between 1 January 2012 and 31 December 2022 were included in this study. The search used a combination of keywords indicating NTM species, patient characteristics, and desired outcome data: (‘nontuberculous mycobacterial infection’ or ‘non-tuberculous mycobacterial infection’ or ‘non-tuberculous mycobacterium’ or ‘nontuberculous mycobacterium’ or ‘*Mycobacterium abscessus*’ or ‘*Mycobacterium massiliense*’ or ‘abscessus abscessus’ or ‘*Mycobacterium fortuitum* complex’ or ‘*Mycobacterium chelonae*’ or ‘*Mycobacterium avium*-intracellulare complex’ or ‘Mycobacterium avium’ or ‘*Mycobacterium intracellulare*’ or ‘*Mycobacterium chimaera*’ or ‘*Mycobacterium kansasii*’ or ‘*Mycobacterium simiae*’ or ‘*Mycobacterium malmoense*’ or ‘*Mycobacterium xenopi*’ or ‘*Mycobacterium genavense*’) and (‘chronic obstructive pulmonary disease’ or ‘COPD’ or ‘asthma’ or ‘bronchiectasis’ or ‘non-CF bronchiectasis’ or ‘non-cystic fibrosis bronchiectasis’) and (‘treatment’ or ‘disease burden’ or ‘clinical outcomes’). The Chinese translation of NTM species followed the Expert Consensus on Chinese Translation of NTM Species,^15^ and the translation of diseases corresponded to Chinese guidelines.^16–18^

### Study selection

The screening process was guided by the inclusion and exclusion criteria based on the Population, Intervention, Comparison, Outcome, and Study (PICOS) framework (Supplementary Table S1). In brief, the search focused on original studies that reported treatment outcomes in Chinese adult patients (≥14 years of age) with NTM-PD and were published in Chinese between 2012 and 2022. The records were first screened by title and abstract. Two reviewers independently performed the selection process recommended by the PRISMA 2020 guidelines for reporting systematic reviews.^14^ The first reviewer performed a comprehensive selection, while the second reviewed and validated the screened studies. Two studies focusing on the effect of traditional Chinese medicine on NTM-PD were excluded to ensure comparability with other global reviews. Any disagreements were jointly discussed and resolved.

### Data extraction and synthesis

The treatment outcome definitions stated in the Non-tuberculous Mycobacteria Network European Trials group (NTM-NET) consensus were adopted for this review (Supplementary Table S2).^19^ Additionally, some studies reporting microbiological outcomes and changes in pulmonary lesions on computed tomography (CT) scans also followed the Chinese guidelines for TB diagnosis and treatment (Supplementary Table S3).^20^

### Ethical approval

Not applicable as this was a systematic literature review.

## RESULTS

### Study attrition and characteristics

**Figure 2.**
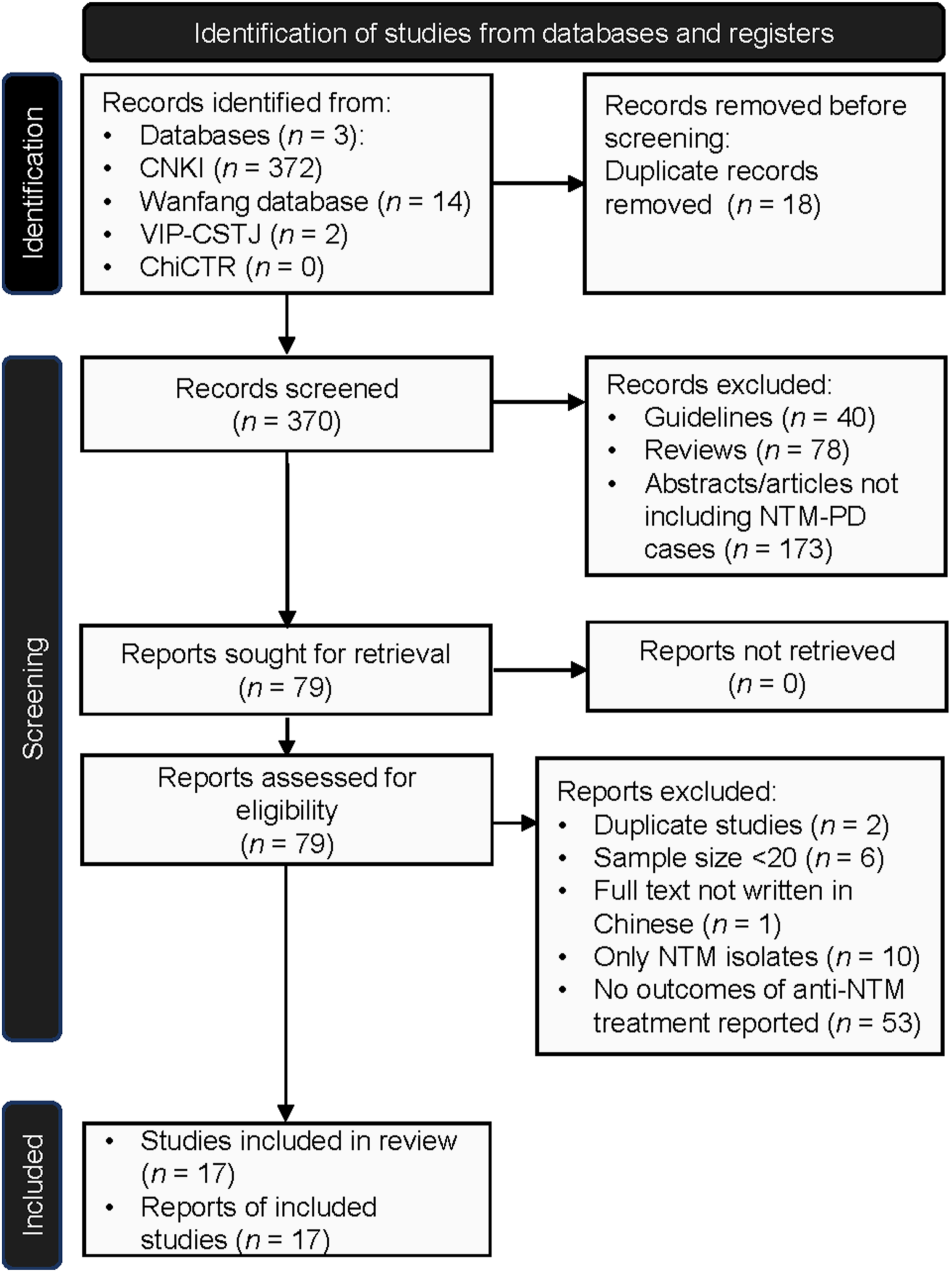
Flow chart of studies included in the systematic review. CNKI = China National Knowledge Infrastructure; VIP-CSTJ = Chinese Science and Technology Journals Database; ChiCTR = Chinese Clinical Trial Register; NTM-PD = NTM pulmonary disease; NTM = non-tuberculous mycobacteria.

A total of 388 records were identified via the CNKI, Wanfang, and VIP-CSTJ databases. After removing 18 duplicate records, 370 records were screened for eligibility. On further screening, 40 guidelines, 78 reviews, and 173 abstracts/articles were excluded for not including NTM-PD cases. A total of 79 articles were reviewed for full-text screening. The remaining records were screened based on the inclusion criteria, leaving 17 articles eligible for this review (Figure 2).

Among the 17 selected studies, 15 were retrospective, while the remaining two studies^21,22^ were prospective randomised controlled trials (RCTs). Within the 15 retrospective studies, 11 studies^23–33^ reported outcomes at the end of treatment (EOT) for patients treated for ≥12 months, and four studies^34–37^ reported the efficacy indicators observed before EOT (Supplementary Table S4).

The majority of patients with NTM-PD are typically middle-aged or elderly. Male patients were slightly less common in most of the studies. Geographically, all the selected studies were conducted in southern China, mainly in the coastal regions. All the studies were conducted in TB-designated hospitals or infectious disease hospitals. Upon diagnosis, patients with NTM-PD are usually suspected of having TB and, as such, are required to move to specially designated hospitals in China’s healthcare system.

The most frequent underlying pulmonary diseases reported in these studies were bronchiectasis (prevalence 31.3–90.7%),^21,22,25,26,28,30,31,34^ COPD (prevalence 27.0–37.8%),^23,24,33,36,37^ and prior exposure to TB (prevalence 16.7–75.0%)^27,29,32,35^ (Supplementary Table S4). All the studies utilised mycobacterial culture of sputum or bronchoalveolar lavage samples to confirm NTM infection. Diagnosis of NTM-PD in all included studies was as per consistent criteria in the Chinese guidelines^11^ and/or ATS guidelines for NTM-PD.^12,13^

### Outcomes at end of treatment

**Figure 3.**
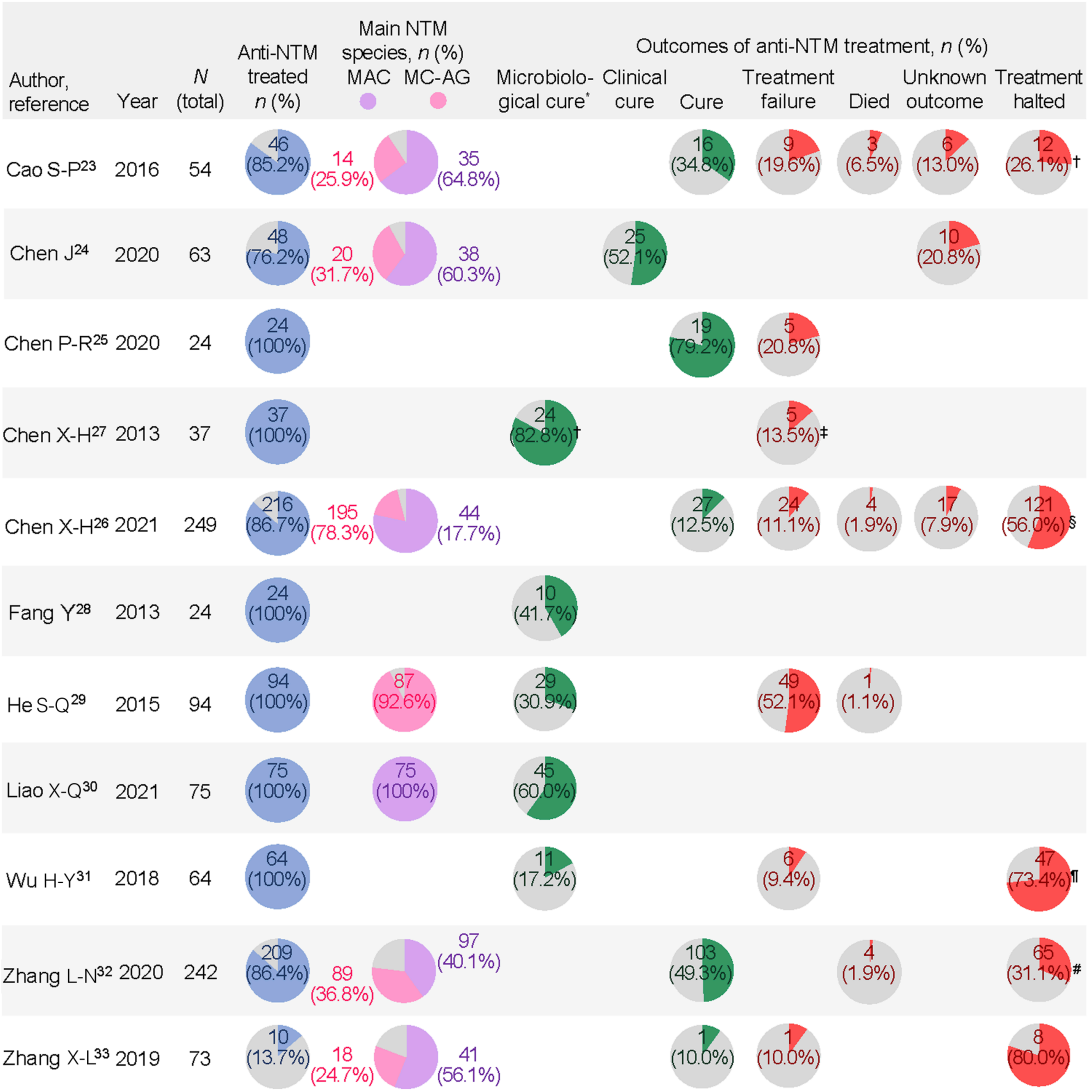
Outcomes at end of treatment in NTM-PD. Data that were not reported are left blank. The proportion of patients with a certain outcome is calculated by dividing the number of patients with that outcome by the total number of patients treated. ^*^The definition of culture conversion was explicitly defined only in the study by Liao et al.,^30^ as the finding of at least two consecutive negative mycobacterial cultures from respiratory samples collected at least 4 weeks apart. ^†^Discontinuation due to intolerance was 33.3% (4/12). ^‡^Out of 29 patients who already completed the therapy at the time of follow-up. ^§^Discontinuation due to intolerance was 63.6% (77/121). ^¶^Discontinuation due to intolerance was 83.0% (39/47). ^#^Discontinuation due to intolerance was 49.2% (32/65). NTM = non-tuberculous mycobacteria; MAC = *Mycobacterium avium-intracellulare* complex; MC-AG = *Mycobacterium chelonae-abscessus* group.

It was noted that a high proportion of patients with NTM-PD received treatment, of which 10.0–79.2% were cured. High variability in sample size could have contributed to a wide range of cure rates across studies. Lower and more consistent microbiological cure rates of 17.2–60.0% were reported in studies with ≥50 patients treated for NTM-PD. Studies with older adults (>60 years) or patients with malnutrition (body mass index <18.5) also showed lower cure/microbiological rates regardless of species distribution.^33,36^ Treatment discontinuation was primarily due to intolerance (footnotes Figure 3). Additionally, prolonged treatment duration, higher medical costs, and non-compliance increased the risk of discontinuation. The outcomes observed at EOT are presented in Figure 3.

### End-of-treatment outcomes differ between MAC and MC-AG infections

Among the 17 selected studies, seven reported the distribution of individual NTM species in patients with NTM-PD (Figure 3).^23,24,26,29,30,32,33^ In line with previous reports, MAC and *Mycobacterium chelonae* abscessus group (MC-AG) were observed to be the two predominant species. Among the seven selected studies, one study^30^ exclusively reported patients infected with MAC, and one study^29^ exclusively reported patients infected with MC-AG. Patients infected with MAC have a higher microbiological cure rate than those with MC-AG infections (60% and 30.9%, respectively) (Figure 3). Overall, better outcomes were observed in studies with a higher proportion of patients infected with MAC than those infected with MC-AG, suggesting more favourable outcomes associated with infection with MAC species than MC-AG. None of the studies reported treatment outcomes by individual NTM species.

### EOT outcomes by individual treatment options

Due to limited disclosure about treatments in the selected studies, no single effective agent was identified. Only one study^30^ reported outcomes by individual regimens, while the others only described the general principles of deciding the antimicrobial combination. This study included patients infected with MAC exclusively and a treatment regimen consisting of clarithromycin (CLM), rifampicin (RIF), and ethambutol (EMB). The addition of amikacin (AMK) or levofloxacin (LFX) to this backbone regimen demonstrated additive effects and increased microbiological cure rates, although without statistical significance (45.5% for CLM + RIF + EMB, 65.7% for CLM + RIF + EMB + AMK, 55.6% for CLM + RIF + EMB + LFX, and 78.5% for CLM + RIF + EMB + AMK + LFX, *P* = 0.359).

**Figure 4.**
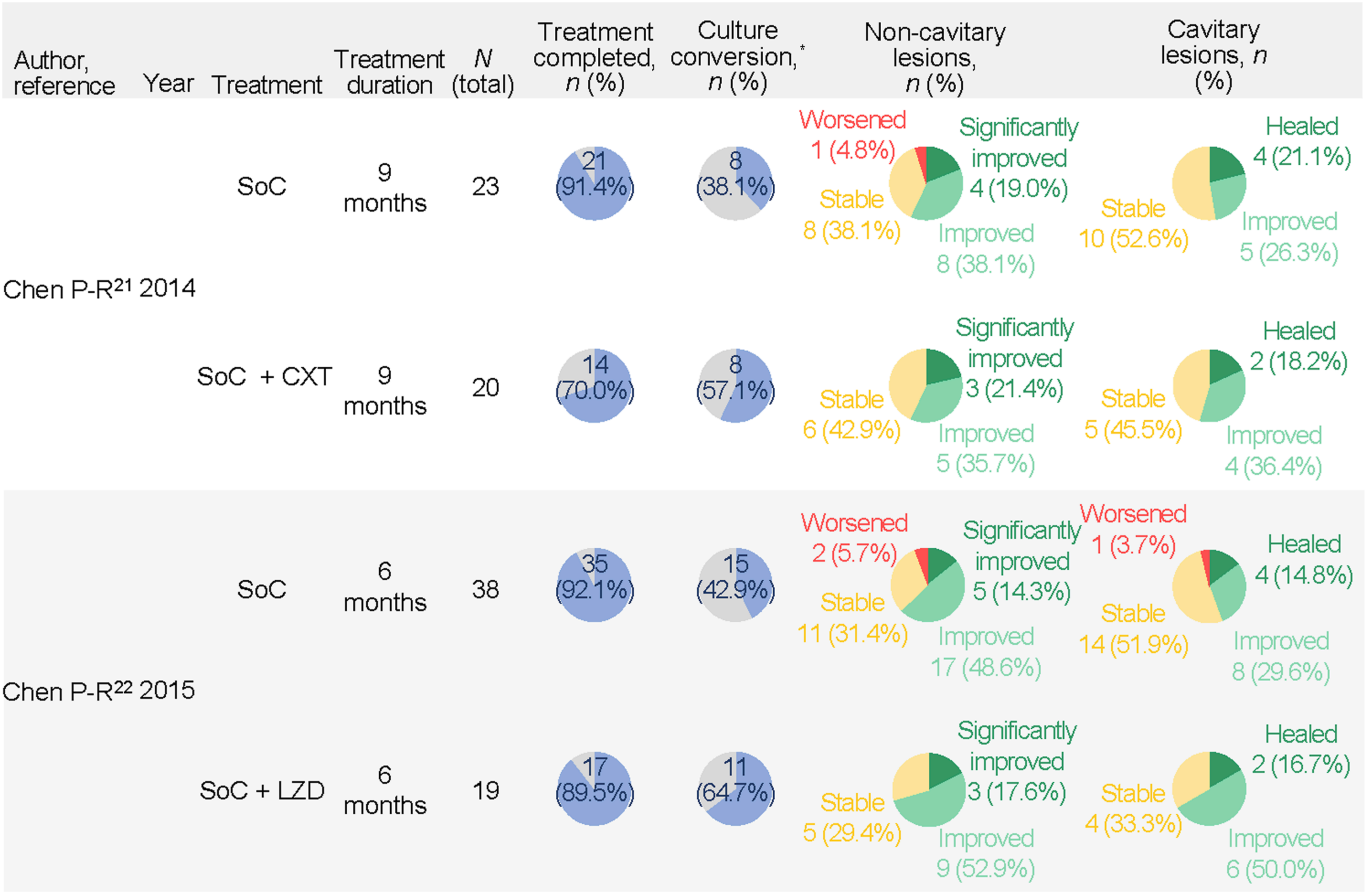
Outcomes with adjunctive CXT or LZD in NTM-PD. SoC is the combined therapy with clarithromycin/azithromycin, amikacin, and 1–2 other antibiotics based on previous treatment and drug susceptibility testing results. The proportion of culture conversion and non-cavitary lesion outcomes was calculated by dividing the number of patients with culture conversion and non-cavitary lesions by the number of patients who completed the treatment; the proportion of cavitary lesion outcomes was calculated by dividing the number of patients with cavitary lesion outcomes by the number of patients with pulmonary cavities.^*^Culture conversion defined as at least three consecutive negative mycobacterial cultures from respiratory samples, collected at least 4 weeks apart. SoC = standard of care; CXT = cefoxitin; LZD = linezolid.

Two RCTs evaluated the effectiveness of adding adjunctive cefoxitin (CXT) and linezolid (LZD) to the treatment regimen (Figure 4).^21,22^ Patients infected with MC-AG were treated with adjunctive CXT (6.0–9.0 g intravenously twice or thrice a day for 2–3 months) combined with the standard of care (SoC, CLM/azithromycin, AMK, and one to two other antibiotics for 9 months). Higher culture conversion rates with CXT + SoC treatment were consistently observed at 2, 6, and 9 months of treatment, although without statistical significance (overall culture conversion: 8/14, 57.1% vs 8/21, 38.1%; *P* = 0.268). Similarly, treatment with adjunctive LZD (600 mg/d intravenously for 2–4 weeks and sequentially 300–600 mg/day orally for a total of 3–6 months) combined with SoC (CLM/azithromycin, AMK, and 1–2 other antibiotics for 9 months) was evaluated in patients with NTM-PD (66.7% rapidly growing mycobacteria and 33.3% MAC). A trend of better culture conversion rates was observed with the addition of LZD (11/17, 64.7% vs 15/35, 42.9%; *P* = 0.060). Both RCTs reported the radiological changes in pulmonary lesions on CT scans but observed no apparent change by adding CXT or LZD to the treatment. Overall, the CT imaging findings remained stable or showed a slight improvement in size after 6–9 months of treatment.

One study^29^ evaluated the outcomes of adjuvant surgical treatment in patients with NTM-PD. Indications for surgery included poor response to drug therapy demonstrated by persistent positive sputum culture (23/24, 95.8%), destructive cavitary lesions (17/24, 70.8%), and/or severe bronchiectasis (23/24, 95.8%) and haemoptysis (16/24, 66.7%), or a combination of these conditions. Patients were given 3–18 months of pre-operative chemotherapy and continued treatment post-operation for at least 12 months. About 79.2% (19/24) of patients achieved cure, defined as sustained negative sputum culture, minimal or no clinically relevant symptoms, and stable lesions on CT scans. No post-operative deaths were observed. However, 8/24 (33.3%) patients experienced post-operative complications, including chest infections (5 patients), caseous necrosis of the lobular stump (2 patients), and poor wound healing (1 patient).

### Outcomes observed while undergoing treatment

**Figure 5.**
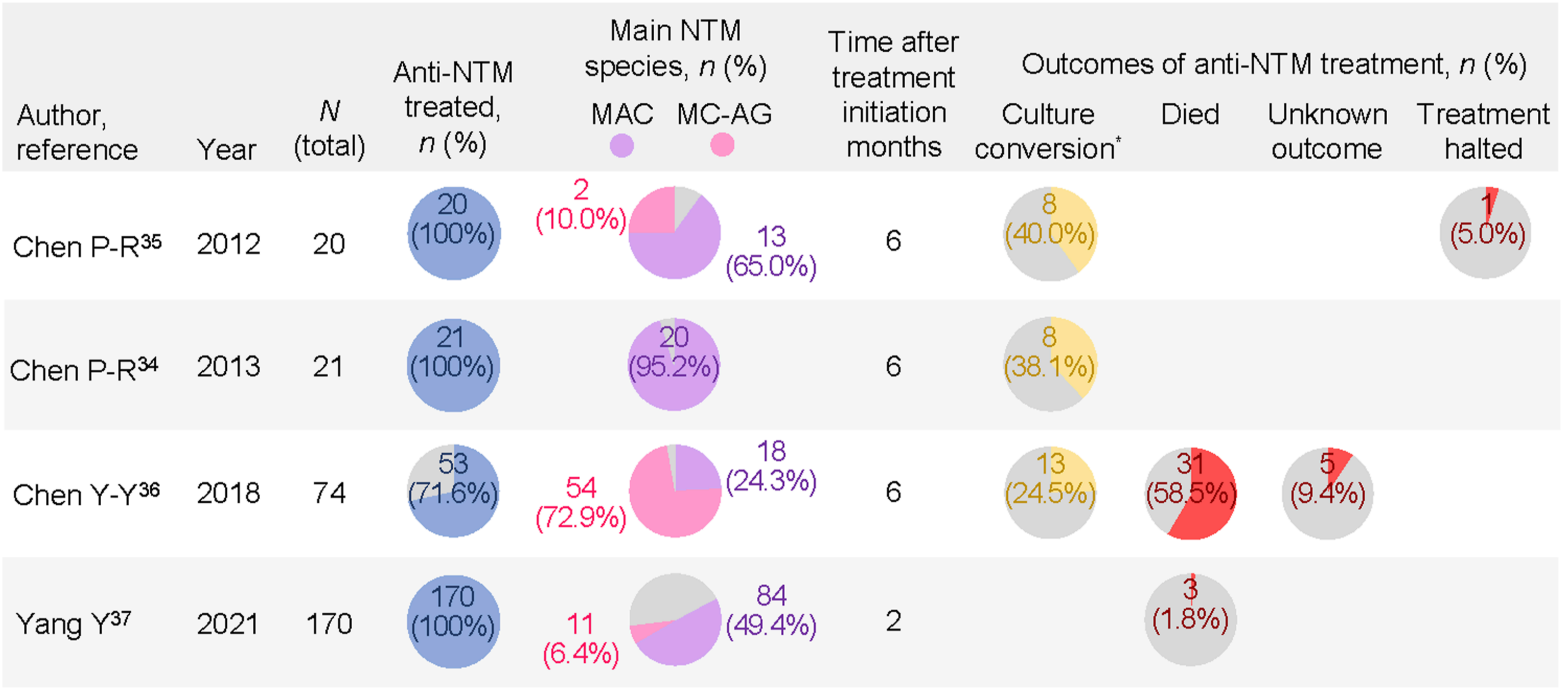
Outcomes while on treatment in NTM-PD. Data that were not reported are left blank. The proportion of patients with a specific outcome was calculated using the number of treated patients as denominator. ^*^Culture conversion defined as in the study by Chen et al.,^35^ i.e., at least three consecutive negative mycobacterial cultures from respiratory samples, collected at least 4 weeks apart. NTM = non-tuberculous mycobacteria; MAC = *Mycobacterium avium-intracellulare* complex; MC-AG = *Mycobacterium chelonae-abscessus* group.

Four studies reported the observed outcomes while patients were undergoing treatment (Figure 5).^34–37^ The culture conversion rates ranged from 24.5% to 40% 6 months after treatment initiation. One study^36^ focused on patients with NTM-PD admitted into intensive care units, mostly (>90%) due to respiratory failure. The patients in this study had a mean age of 74.7 ± 8.7 years, and the prominent species detected were *M. abscessus* (54/74, 72.9%) and *M. intracellulare* (16/74, 21.6%). The observed case fatality rate was high (58.5%), possibly due to combined host and pathogen risk factors.

## DISCUSSION

This systematic review of Chinese-language publications on treatment outcomes in NTM-PD identified 17 eligible studies. We found that the current standard of care (SoC), involving combined antimicrobial therapy recommended in Chinese guidelines, led to unsatisfactory treatment outcomes. Increased age and infections with the MC-AG were associated with poorer outcomes. However, adjunctive surgical treatment combined with SoC showed a cure rate of 79.2% in a small group of patients, albeit with a complication rate of 33.3%.

A high prevalence of TB (16.7–75.0%) among patients with NTM-PD was also reported. Patients with prior TB are at an increased risk of developing NTM-PD, likely due to TB-related bronchiectasis and cavitation sequelae.^38,39^ Persistent structural damage from TB may contribute to a fibrocavitary phenotype in subsequent NTM infection, resulting in unfavourable outcomes. Some patients with a history of TB may have been misdiagnosed and only identified with NTM due to treatment failure or disease recurrence.^40^ Delayed diagnosis and treatment of NTM-PD also contribute to poorer outcomes. This high TB burden could be linked to the slightly lower sputum culture conversion rates seen in Chinese patients, underscoring the importance of distinguishing between TB and NTM-PD.

One study in our review reported a cure rate of 79.2% in patients receiving adjunctive surgery for NTM-PD, though these results should be interpreted cautiously. Surgery combined with chemotherapy may enhance outcomes by removing lesions harbouring germ reservoirs, such as cavitary lesions and caseous necrosis. Careful consideration of lung surgical resection could serve as an adjunct to medical therapy in selected patients.^41^ Typically, surgery is reserved for patients with persistent positive cultures after >12 months of antibiotic treatment, cavitary disease, drug-resistant isolates, or specific complications.^41^ Several American and Japanese studies^42–44^ have shown superior post-operative negative culture rates with surgery combined with chemotherapy, but the long-term benefits remain unclear. While surgery shows promise, it is generally limited to patients with disease confined to one or two lobes, good post-operative lung function, and tolerance for surgery and anaesthesia. Moreover, most studies assessing surgical outcomes were retrospective, single-centre, and involved a selected population, introducing potential bias. More robust evidence is needed to establish the role of adjunctive surgery in NTM-PD treatment.

A promising treatment strategy is the repurposing of readily available antimicrobials for NTM-PD, focusing on candidates effective against both MAC and MABC species. LZD is a potent antimicrobial used for multidrug-resistant TB and is active against many NTM species.^45^ In China, susceptibility rates to LZD are 14.8% for MAC and 32.46% for MABC,^7,46^ compared to around 13% for MAC and 29% for MABC in the European Union and the US.^45,47,48^ LZD as an adjunctive drug in NTM-PD treatment has been associated with better outcomes compared to SoC.^22^ Despite some reports of therapeutic success,^49,50^ LZD carries treatment-limiting adverse events such as cytopenia and neuropathy. A retrospective study (*n* = 102) showed that more than 40% of patients with NTM-PD experienced LZD-related adverse events, leading to drug discontinuation at a median of 20 weeks (range: 1–109 weeks).^51^ In the RCT^22^ included in our review, 52.9% of patients experienced adverse events, with only 5.3% discontinuing treatment in the LZD group, possibly due to the lower LZD dose (300–600 mg) used after 2–4 weeks. However, it remains unclear whether doses below 600 mg provide clinical benefits in NTM-PD. More evidence is needed on the risks and benefits of long-term LZD use in NTM-PD.

CXT has demonstrated potent antibacterial activity against rapidly growing mycobacteria in vitro and is recommended by the ATS/IDSA for MABC infections. However, the susceptibility rates to CXT in MABC range from 0% to 40% in mainland China^52–54^ and 0% to 27% in the European Union and the United States.^48^ The RCT^21^ comparing adjunctive CXT + SoC with SoC alone in MC-AG pulmonary diseases showed some benefits with adjunctive CXT, although these were not statistically significant. The small sample size, limited 9-month treatment duration, and lack of differentiation between MC-AG infections contributed to the inconclusive data. This is consistent with a meta-analysis that found no association between CXT use and treatment success in *M. abscessus* pulmonary disease.^55^ Future in vitro and clinical studies are necessary to identify other effective antimicrobials for NTM-PD.

This review has several limitations, including potential biases in interpreting outcomes due to inconsistent definitions and the small number of studies reviewed. The lack of consensus on treatment outcome definitions and guidelines over the years has likely led to inconsistent reporting of outcomes. Additionally, most studies did not provide clear definitions of the outcomes they used, which reduced comparability across studies. Long-term outcomes of NTM-PD could not be evaluated, as most studies did not report data beyond EOT. The wide range (10.0–82.8%) of microbiological cure rates also limits the significance of this review. It was challenging to identify consistent patterns of patient characteristics that could help determine risk factors for lower microbiological cure rates. This may be attributed to the small sample sizes in some studies, the limited number of studies included, heterogeneous patient characteristics, lack of species-specific outcome data, or potentially inconsistent outcome definitions. Consequently, the observations from the included studies may serve more as qualitative insights. Since all the studies were conducted in China’s southern and coastal regions, the observed predominance of MAC-PD and MABC-PD with suboptimal outcomes may not be generalisable to other regions. Nevertheless, these limitations highlight the prevalence and importance of NTM-PD in China and underscore the need for more standardised treatment protocols, long-term follow-up, and reporting practices to generate more robust real-world evidence and improve patient management.

## CONCLUSION

This is the first systematic review of Chinese-language publications on NTM-PD, providing a comprehensive overview of NTM-PD morbidity and management across multiple endemic provinces in southern and coastal China. This review offers updated information to the international research community and complements earlier studies, which were mostly limited to single provinces. The prevalence of NTM-PD in China is rising, primarily due to MAC and MABC. However, the current treatment regimen recommended in Chinese guidelines achieves only moderate treatment success rates and presents poor safety profiles for Chinese patients with NTM-PD. The findings from this review can help inform future NTM-PD treatment strategies tailored to bacterial and host factors to optimise patient outcomes.

## Supplementary Material


