# Supplementary material for: Treatment outcomes in NTM-PD in a high TB burden context
Source: IJTLD Open. 2024 Dec 1;1(12):547–55. doi: 10.5588/ijtldopen.24.0413 (PMC11636495; doi:10.5588/ijtldopen.24.0413)
Supplement: Supplementary file 1 [file ijtldopen24-0413_supplementarydata1.pdf]

http://dx.doi.org/10.5588/ijtldopen.24.0413

Treatment outcomes in NTM-PD in a high TB burden context

Supplementary Table S1: Study eligibility criteria defined by the PICOS criteria

Items	Inclusion criteria	Exclusion criteria
**Population**	Adult patients with NTM-PD (≥14 years of age)	Patients <14 years of age
**Intervention**	Any, none required	No exclusion based on ‘intervention’
**Comparators**	Any, none required	No exclusion based on ‘comparator’
**Outcomes of interest**	Treatment outcomes and complications of NTM-PD	Studies that do not report on treatment outcomes and complications associated with NTM-PD
**Study design**	Original studies	Original studies with only NTM strain data or <20 patient cases<br>All other types of studies were excluded (including review articles, conference abstracts, commentaries, and editorials)
**Location**	China	Countries except China
**Language**	Chinese	All other languages
**Year**	Studies published between 2012 and 2022	Years before 2012<br>Years after 2022

NTM: nontuberculous mycobacteria; NTM-PD: nontuberculous mycobacterial pulmonary disease; PICOS: Population, Intervention, Comparison, Outcome, and Study.

Supplementary Table S2: Treatment outcome definitions as per the NTM-NET consensus [15]

Outcome parameter	Definition
**Culture conversion**	The finding of at least three consecutive negative mycobacterial cultures from respiratory samples, collected at least four weeks apart, during antimycobacterial treatment (the sampling date of the first negative culture is then the date of culture conversion)
**Microbiological cure**	Finding multiple consecutive negative and no positive cultures with the causative species from respiratory samples after culture conversion and until the end of antimycobacterial treatment
**Cure**	Antimycobacterial treatment completed, with fulfilment of criteria for both microbiological and clinical cure
**Clinical cure**	Patient-reported and/or objective improvement of symptoms during antimycobacterial treatment, sustained until at least the end of treatment, but no cultures available to prove culture conversion or microbiological cure
**Treatment failure**	The re-emergence of multiple positive cultures or persistence of positive cultures with the causative species from respiratory samples after ≥12 months of antimycobacterial treatment, while the patient is still on treatment
**Recurrence**	The re-emergence of at least two positive cultures with the causative species from respiratory samples after cessation of antimycobacterial treatment
**Relapse**	The emergence of at least two positive cultures with the same strain of the causative species after the end of treatment
**Reinfection**	The emergence of at least two positive cultures with a different strain of the causative species or a strain of a different species after the initiation of the treatment episode
**Died**	Death due to any reason, but during NTM-PD treatment
**Unknown outcome**	Patient is no longer seen by his/her treating physician, so follow-up of treatment outcome is not possible (umbrella term for "lost to follow-up" and "transfer out")
**Died due to NTM-PD**	All causes of death that would not have occurred if the patient had not had NTM-PD
**Treatment halted**	Physician- or patient-initiated pre-term cessation of antimycobacterial treatment

NTM: nontuberculous mycobacteria; NTM-NET: Nontuberculous Mycobacteria Network European Trials group; NTM-PD: nontuberculous mycobacterial pulmonary disease.

Supplementary Table S3: Treatment outcome definitions as per Chinese guidelines for TB diagnosis and treatment

Outcome parameter	Definition
**Culture conversion**	The finding of at least three consecutive negative mycobacterial cultures from respiratory samples, collected at least four weeks apart
**Non-cavitary lesions on CT scan**	Significantly improved: the size of the lesions decreased by ≥1/2
	Improved: the size of the lesions decreased by <1/2
	Stable: no obvious changes in lesion size
	Worsened: the lesions increased in size or distribution
**Cavitary lesions on CT scan**	Healed: no cavities observed
	Improved: the cavitary lesions decreased by ≥1/2 in diameter
	Stable: the cavitary lesions changed by <1/2 in diameter
	Worsened: the cavitary lesions increased by >1/2 in diameter

CT: computerized tomography; TB: tuberculosis.

Supplementary Table S4: Study characteristics

Author and reference	Year	Region[a]	Clinical setting	N (total)	Male, n (%)	Age (years)	Enrolling period	Method of NTM diagnosis	Diagnostic criteria for NTM-PD	Most common underlying pulmonary disease, n (%)
**Studies with EOT outcomes**										
**Cao Shi-peng et al.**	2016	Southern/Inland	Inpatients from TB-designated hospital	54	34 (63.0)	Mean: 58.8±12.2, range: 21–85	01/2013–12/2013	Sputum or BAL culture	2012 Chinese guidelines	COPD, 18 (33.3)
**Chen Jia et al.**	2020	Southern/Inland	Inpatients from TB-designated hospital	63	27 (42.9)	Mean: 49.3±18.5, range: 15–83	01/2016–12/2017	Sputum culture	2012 Chinese guidelines	COPD, 17 (27.0)
**Chen Pin-ru, et al.**	2020	Southern/Coastal	From TB-designated hospital	24	9 (37.5)	Median: 44.5, quartile: 30.8, 55.0	01/2009–12/2017	Sputum culture	2012 Chinese guidelines	Bronchiectasis, 23 (95.8)
**Chen Xiao-hong et al.**	2013	Southern/Coastal	Inpatients from TB-designated hospital	37	16 (43.2)	Median: 56.6, range: 21–72	01/2008–10/2011	Sputum or BAL culture	2012 Chinese guidelines	Previous TB, 6 (16.7)
**Chen Xiao-hong et al.**	2021	Southern/Coastal	Inpatients from TB-designated hospital	249	116 (46.6)	Mean of males: 61.9±11.6, mean of females: 59.8±12.3	01/2018–01/2020	Sputum or BAL culture	2020 Chinese guidelines	Bronchiectasis, 175 (70.3)
**Fang Yong et al.**	2013	Southern/Coastal	From TB-designated hospital	24	10 (41.7)	Mean: 56.0±11.0, range: 32–	01/2007–12/2009	Sputum culture	2000 Chinese guidelines	Bronchiectasis, 12 (50.0)

Author and reference	Year	Region[a]	Clinical setting	N (total)	Male, n (%)	Age (years)	Enrolling period	Method of NTM diagnosis	Diagnostic criteria for NTM-PD	Most common underlying pulmonary disease, n (%)
						76				
**He Si-qi et al.**	2015	Southern/Coastal	From TB-designated hospital	94	37 (39.4)	Mean: 50.9±3.7, range: 17–84	01/2008–06/2014	Sputum or BAL culture	2012 Chinese guidelines + 2007 ATS guidelines	Previous TB, 57 (60.6)
**Liao Xiao-qin et al.**	2021	Southern/Coastal	Inpatients from TB-designated hospital	75	34 (45.3)	Range: 24–90	10/2017–12/2018	Sputum or BAL culture	2020 Chinese guidelines	Bronchiectasis, 57 (76.0)
**Wu Hai-yan et al.**	2018	Southern/Coastal	From infectious disease hospital	64	38 (59.4)	Median: 54	09/2014–09/2016	Sputum culture	2012 Chinese guidelines	Bronchiectasis, 20 (31.3)
**Zhang Li-na et al.**	2020	Southern/Coastal	From infectious disease hospital	242	101 (41.7)	Mean: 49.5± 15.3, range: 14–86	11/2012–07/2017	Sputum or BAL culture	2012 Chinese guidelines	Previous TB, 57 (23.6)
**Zhang Xi-lin et al.**	2019	Southern/Coastal	From TB-designated hospital	73	35 (47.9)	Mean: 62.6±14.3, range: 24–86	01/2016–07/2017	Sputum culture	2012 Chinese guidelines	COPD, 23 (31.5)

Author and reference	Year	Region[a]	Clinical setting	N (total)	Male, n (%)	Age (years)	Enrolling period	Method of NTM diagnosis	Diagnostic criteria for NTM-PD	Most common underlying pulmonary disease, n (%)
**Studies with on-treatment outcomes**										
**Chen Pin-ru et al.**	2012	Southern/Coastal	From TB-designated hospital	20	8 (40.0)	Mean: 54.5±14.3, range: 28–73	01/2008–10/2010	Sputum culture	2000 Chinese guidelines	Previous TB, 15 (75.0)
**Chen Pin-ru et al.**	2013	Southern/Coastal	From TB-designated hospital	21	7 (33.3)	Mean: 54.0±16.0, range: 21–73	01/2008–10/2011	Sputum or BAL culture	2000 Chinese guidelines	Bronchiectasis, 18 (85.7)
**Chen Yuanyuan et al.**	2018	Southern/Coastal	ICU inpatients from TB-designated hospital	74	55 (74.3)	Mean: 76.5±8.7	01/2012–05/2017	Sputum or BAL culture	2012 Chinese guidelines	COPD, 28 (37.8)
**Yang Yan et al.**	2021	Southern/Coastal	Inpatients from infectious disease hospital	170	86 (50.6)	Mean: 61.9±2.4	1/2018–12/2019	Sputum culture	2020 ATS guidelines	COPD, 56 (32.9)
**RCTs**										
**Chen Pin-ru et al.**	2014	Southern/Coastal	From TB-designated hospital	SoC[b]: 23, SoC+Cxt: 20	7 (30.4) 5 (25.0)	Mean: 53.5±15.5, mean: 55.7±12.3	1/2008–12/2012	Sputum culture	2012 Chinese guidelines + 2007 ATS	Bronchiectasis, 39 (90.7)

Author and reference	Year	Region[a]	Clinical setting	N (total)	Male, n (%)	Age (years)	Enrolling period	Method of NTM diagnosis	Diagnostic criteria for NTM-PD	Most common underlying pulmonary disease, n (%)
									guidelines	
**Chen Pin-ru et al.**	2015	Southern/Coastal	From TB-designated hospital	SoC[b]: 38, SoC+LZD: 19	12 (31.6) 4 (21.1)	Mean: 53.5±15.5, mean: 46.3±14.6	1/2008–6/2014	Sputum culture	2012 Chinese guidelines + 2007 ATS guidelines	Bronchiectasis, 51 (89.5)

Note: data that were not reported in individual studies were left blank
ATS, American Thoracic Society; BAL, bronchoalveolar lavage; COPD, chronic obstructive pulmonary disease; Cxt, cefoxitin; EOT: end of treatment; ICU, intensive care unit; LZD, linezolid; N, number; NTM, nontuberculous mycobacteria; NTM-PD, nontuberculous mycobacterial pulmonary disease; RCTs, randomized controlled trials; SoC, standard of care; TB, tuberculosis.

[a]The 31 provinces of mainland China are divided both longitudinally and latitudinally.
Provinces located in northern region: Beijing, Gansu, Hebei, Heilongjiang, Henan, Inner Mongolia, Jilin, Liaoning, Ningxia, Qinghai, Shaanxi, Shandong, Shanxi, Tianjin, Tibet, Xinjiang.
Provinces located in southern region: Anhui, Chongqing, Fujian, Guangdong, Guangxi, Guizhou, Hainan, Hubei, Hunan, Jiangsu, Jiangxi, Shanghai, Sichuan, Yunnan, Zhejiang.
Provinces located in coastal region: Fujian, Guangdong, Guangxi, Hainan, Hebei, Jiangsu, Liaoning, Shandong, Shanghai, Tianjin, Zhejiang.
Provinces located in inland region: Anhui, Beijing, Chongqing, Gansu, Guizhou, Heilongjiang, Henan, Hubei, Hunan, Inner Mongolia, Jiangxi, Jilin, Ningxia, Qinghai, Shaanxi, Shanxi, Sichuan, Tibet, Xinjiang, Yunnan.
[b]SoC is the combined therapy with clarithromycin/azithromycin, amikacin, and one to two other antibiotics based on previous treatment and drug susceptibility testing results.

- Chen P-r, Xiao F, Deng Z-x, et al. [The short-term effect of cefoxitin-containing drug regimen in the treatment of M. abscessus pulmonary disease]. Guangdong Medical Journal. 2014;35(11):1760-4. doi: 10.13820/j.cnki.gdyx.2014.11.050.
- Chen P-r, Xiao F, Deng Z-x, et al. Short-term efficacy and safety of linezolid combination therapy non-tuberculous mycobacterial lung disease. Modern Hospital. 2015;15(12):13-7.
- Cao S-p, Fu M-j, Luo D. Analysis on clinical characteristics and diagnosis-treatment experience of 54 cases of nontuberculous mycobacteria pulmonary disease. Guangxi Medical Journal. 2016;38(03):328-31.
- Chen J. Clinical analysis of 63 cases of NTM pulmonary disease. Journal of China Prescription Drug. 2020;18(06):174-7.
- Chen P-r, Tan S-y. The role of surgery in the treatment of non-tuberculous mycobacterium pulmonary disease. Chinese Journal of Antituberculosis. 2020;42(02):159-63.
- Chen X, Liao X, Wu D, Lin J. Clinical characteristics of non-tuberculous mycobacterial pulmonary disease in Fuzhou. Chinese Journal of Infection Control. 2021;20(8):688-93.
- Chen X-h, Wang L, Zheng X-h, et al. Misdiagnosis analysis of 37 cases with pulmonary disease caused by nontuberculosis mycobacteria. Journal of Tuberculosis and Lung Health. 2013(02):119-21.

- Fang Y, Sha W. Efficacy of cefoxitin in treatment of rapidly growing nontuberculous mycobacterial pulmonary disease. Journal of Tongji University (Medical Science). 2013;34(02):51-4.
- He S-q, Chen P-r. An analysis of the clinical features of 94 cases pulmonary disease caused by rapidly growing mycobacteria. The Journal of The Chinese Antituberculosis Association. 2015;37(9).
- Liao X-q, Lin J-d, Wu D, Chen X-h. Analysis of the clinical characteristics of Mycobacterium avian complex pulmonary disease and the risk factors of treatment effect. Journal of Tuberculosis and Lung Disease. 2021;2(02):120-4.
- Wu H-y, Wu M-y. Clinical characteristics analysis of 64 patients with non-tuberculous mycobacterial lung disease in Suzhou. Journal of Clinical Pulmonary Medicine. 2018;23(04):628-31.
- Zhang L-n, Li X, Deng Q-y, et al. Treatment Outcomes of Nontuberculous Mycobacterial Pulmonary Disease and Influencing Factors. Shenzhen Journal of Integrated Traditional Chinese and Western Medicine. 2020;30(08):12-5.
- Zhang X-l, Zhong Y-h, He Z-h, et al. Clinical analysis of 73 patients with non-tuberculous mycobacterial pulmonary disease. Journal of Clinical Pulmonary Medicine. 2019;24(02):212-6.
- Chen P-r, Tan S-y. The treatment outcomes of 21 cases of rapidly growing mycobacteria related pulmonary disease. Chinese Journal of Tuberculosis and Respiratory Diseases. 2013(03):182-5.
- Chen P-r, Xiao F, Chen H, et al. [Efficacy and safety of individualized treatment of non-tuberculous mycobacterial lung disease]. Guangdong Medical Journal. 2012;33(11):1648-51.
- hen Y-y, Mao M-j, Fan D-p, et al. Comparison of clinical features of pulmonary infections with Mycobacterium intracellulare and Mycobacterium abscessus in tuberculosis intensive care unit. Chinese Journal of Clinical Infectious Diseases. 2018;11(03):191-6+212.
- Yang Y, Zeng Y. Retrospective analysis of 170 cases of non-tuberculous mycobacterial pulmonary disease. Journal of Nanjing Medical University(Natural Sciences). 2021;41(07):1058-62.
